# Corporate social responsibility and frontline employees’ service improvisation: The mediating role of self-efficacy

**DOI:** 10.3389/fpsyg.2022.898476

**Published:** 2022-11-18

**Authors:** Xuezhao Zhang, Siyuan Zhang, Mingsen Wang

**Affiliations:** School of Economics and Management, Zhoukou Normal University, Zhoukou, China

**Keywords:** frontline employees, corporate social responsibility, self-efficacy, service improvisation, tourism enterprise

## Abstract

The uncertainty of the COVID-19 pandemic has brought unprecedented challenges to frontline employees in tourism enterprises. In the context of the COVID-19 pandemic, the fulfillment of corporate social responsibility is of great significance. Based on the social cognitive theory, a conceptual framework was established to investigate the relationship between corporate social responsibility and tourism service improvisation, along with the mediating role of self-efficiency. A total of 405 self-administered questionnaires were collected through three times. The results revealed that frontline employees’ perception of corporate social responsibility had a significant positive impact on self-efficacy and service improvisation, as well as self-efficacy had a significant positive impact on service improvisation. Meanwhile, self-efficacy played a partial mediating role in the relationship between corporate social responsibility and service improvisation. Theoretical and practical implications, along with limitations and future research directions, were discussed.

## Introduction

The tourism industry, a high-contact industry, has been hit hard by the outbreak of COVID-19 (Kim and Lee, 2020). As a service industry, the tourism industry has the simultaneity of production and consumption (Rathmell, 1966). The simultaneity has led to frontline employees becoming an important bridge between tourism enterprises and their customers (Jung and Yoon, 2018). The performance of frontline employees in the service process has a direct impact on both tourism companies and customers (Gaur et al., 2017). In order to ensure the service experience of customers, tourism companies generally formulate detailed service standards and processes (Ding and Keh, 2016). However, due to the uncertainty of customer demand, frontline employees are often required to provide satisfactory service to customers according to specific service requirements on the basis of original service standards (Chan et al., 2021). This kind of service behavior of frontline employees that deviates from the original service standard is called service improvisation (Secchi et al., 2019). In the tourism industry, frontline employees often have to deal with the service issues caused by customer uncertainty (Frei, 2006). Many tourism enterprises managers also find it impossible to train frontline employees to deal with all possible situations (Oh and Jang, 2020). In order to continuously meet the needs of customers, relevant scholars have introduced the concept of improvisation into the service industry (Secchi et al., 2019). Service improvisation is a combination of service and improvisation, a special manifestation of the act of improvisation in the service industry. Improvisation is the behavior of an employee who bypasses formal organizational plans to think quickly and act immediately when it is impossible to set aside more time to find a solution (Hadida et al., 2015). Research on improvisation as an unconventional way to cope with an increasingly complex and uncertain business environment is attracting the interest of scholars and industry professionals. Improvisation is not only a source of organizational change and innovation, but also an effective strategy for employees to break through existing cognitive constraints to stimulate innovative thinking and improve job performance (Hadida et al., 2015). At present, the study of employee improvisation is in its infancy (Secchi et al., 2019). Some scholars have explored the role of organizational situational factors such as team cohesion (Magni et al., 2009), organizational culture (Hadida et al., 2015), and organizational memory on the stimulating effect of employees’ improvisation. At the same time, existing research also explores the influencing factors of service improvisation from the perspective of employees’ psychological state, such as employees’ emotional intelligence (Hill et al., 2017), time pressure (Pina e Cunha et al., 2009), and self-efficacy (Yeboah Banin et al., 2016). However, there has been relatively little inquiry into the influencing mechanism of employee service improvisation, especially in the tourism industry (Secchi et al., 2019). In addition, the outbreak of COVID-19 has brought significant uncertainties to the tourism industry, such as the uncertainty of business activities, the uncertainty of customers’ normal travel, and the uncertainty of employees’ physical and mental health. To the best of our knowledge, there is no research on the influencing mechanism of employee service improvisation based on the context of COVID-19. Compared with the past, frontline employees of tourism enterprises face more complex and changeable service situations and physical and mental pressures (Chan et al., 2021). In the context of COVID-19, strengthening the study of the influencing mechanism of frontline employee service improvisation can not only strengthen the company’s attention to the physical and mental health of employees, but also enhance customer service satisfaction, thereby enhancing the ability of tourism enterprises to respond to the epidemic crisis.

Social cognition theory is a theory that studies the environment, psychology and behavior (Bandura, 1995). The theory states that an individual’s behavior is co-influenced by the environment and psychology (Bandura, 1995). Therefore, based on the background of COVID-19, this study mainly explores the influencing mechanism of service improvisation of frontline employees in tourism enterprises from two aspects: external environment and employee psychology.

The catastrophic impact of COVID-19 on the market level has forced tourism enterprises to consider all possible measures to promote recovery and sustainable development (Zhang et al., 2021). Corporate social responsibility (CSR) is a common strategic tool and management practice adopted by enterprises in crisis situations (Ham and Kim, 2020), which has been valued by many tourism enterprises (Zhang et al., 2021). For example, some travel agencies use their own channels to purchase gauze masks, goggles and other protective equipment for frontline employees in the face of a shortage of medical supplies. Many hotels offer free space for medical staff or medical observation. Some tourism enterprises issue medical supplies and living allowances to employees who are quarantined at home. These activities are the concrete embodiment of the fulfillment of social responsibility of tourism enterprises. Corporate social responsibility (CSR) means that the operation of an enterprise should not only consider the interests of shareholders, but also comprehensively consider the interests of other stakeholders (e.g., customers, employees, and the public). As an important external environment exposed to frontline employees, CSR is worth in-depth discussion (Onkila and Sarna, 2022). The existing research on CSR mainly focuses on the macro level, that is, from the perspective of firms or institutions to explore the importance of CSR (Ibarnia et al., 2020). For example, some scholars have verified that CSR is beneficial for improving the image and performance of companies (Zhao and Murrell, 2016). However, the effectiveness of fulfilling CSR performance in response to the crisis is still under debate (Aguinis et al., 2020). Shin et al. (2021) believe that the implementation of CSR can maintain the hotel’s performance and customer bookings during the COVID-19 pandemic. Bae et al. (2021) also discuss the benefits of CSR fulfillment for companies’ stock market recovery during the COVID-19 pandemic. Conversely, some scholars have also questioned the effectiveness of CSR, arguing that the implementation of CSR does not necessarily help companies to respond effectively to the crisis of COVID-19 (Aguinis et al., 2020). However, as an important stakeholder of the company, there are relatively few micro-discussions about CSR from the perspective of employees (Wang et al., 2020). Although scholars have begun to explore the relationship between CSR and employee positive behavior (Glavas and Godwin, 2013; Vlachos et al., 2014), the debate on the impact of CSR on employee work behavior is still fierce (Onyishi et al., 2020). Many scholars have pointed out that the influencing mechanism between CSR and employees’ extra-role behaviors has not been fully studied and solved (Glavas, 2016; Jones et al., 2017; Onyishi et al., 2020). In crisis situations, a good CSR reputation has the effect of buffering risks and mitigating crisis damage (Zhang et al., 2021), which assists tourism enterprises in maintaining a good corporate image, and achieving the recognition and support from internal and external stakeholders. Therefore, in the context of the COVID-19 pandemic, it is crucial to study the influence of CSR on frontline employees service improvisation in tourism enterprises.

Frontline employees, as the bridge connecting tourism enterprises and customers, are often faced with greater work pressure. Therefore, research on the psychological state of frontline employees has always been the focus of scholars. Scholars have explored the psychological state of employees in terms of time pressure (Sijbom et al., 2018), emotioncy (Pishghadam and Abbasnejad, 2017), psychological empowerment (Secchi et al., 2019) and so on. For example, the concept of emotioncy (emotion + frequency) was first introduced by Pishghadam et al. (2013). The emotioncy notion presents that “individuals can construct their idiosyncratic understanding of the world through their senses” (Pishghadam and Abbasnejad, 2017). As an important psychological state, self-efficacy is of great significance for employees to cope with uncertainty, especially in the time of COVID-19 (Tang et al., 2020). Self-efficacy (SE) is an individual’s belief, judgment, or subjective perception of the level at which he or she can complete a behavioral activity before performing that behavioral action (Bandura, 1995). Prior to the action, the individual will make a competent speculation and judgment about whether he can carry out a certain action. A high level of SE occurs when an individual is convinced of his or her ability to perform a particular task successfully. In general, frontline employees are under pressure at work, and SE can effectively alleviate the negative impact of stress on individuals (Schaubroeck et al., 2000). When frontline employees have lower self-efficacy, they experience intense anxiety because they hold doubts about their ability to deal with and control potential threats to their environment. In this case, they may see difficulties as more serious than they really are and seriously doubt their own abilities, thus deterring them from taking action (Siu et al., 2005). Conversely, employees with higher SE tend to use aggressive coping strategies. They see the challenges and stresses at work as a great opportunity to learn a variety of new skills. They do not shrink back or give up in the face of difficulties, which in turn alleviates the negative impact of stress on themselves (Stumpf et al., 1987). In a word, SE makes employees more confident to engage in certain activities and more sensitive to relevant information in the environment. They take the initiative to find solutions to problems and persist longer in the face of difficulties, setbacks, and failures (Tang et al., 2020). The importance of SE has been recognized. Numerous scholars have pointed out that SE can significantly affect employee performance (Cherian and Jacob, 2013; Zhang et al., 2020). Some scholars have also argued that SE has a positive impact on employees’ organisational citizenship behavior (Ullah I. et al., 2021). In addition, a few scholars have also found a anegative association between SE and employee behavior (Vancouver and Kendall, 2006; Lin et al., 2016). There has been relatively little research on the influencing factors of SE, focusing mainly on leadership styles (Newman et al., 2019). Existing research has also confirmed that SE plays an important mediating role between environmental and personal factors (Abdullah and Marican, 2020; Tang et al., 2020). However, there is little literature examining SE of frontline employees in high-pressure contexts (Shao et al., 2019).

In a word, this study will discuss the influencing mechanism between CSR and SI of frontline employees in tourism enterprises, and clarify whether and how SE can play an important mediating role between CSR and SI in the context of the COVID-19 pandemic.

## Literature review and hypotheses

### Social cognitive theory

Social cognitive theory (SCT) is widely used in the study of organizational behavior (Kim and Beehr, 2017). The theory holds that individuals are not only influenced by the external environment, but also by their own internal factors (Bandura, 2012). On the basis of acknowledging the subjective initiative of individuals, SCT systematically reveals the process of generating individual behavior from the perspective of individual cognition (Bandura, 2001). The basic assumption of SCT is that there is an ongoing interaction between the external environment, individual cognition and individual behavior (Bandura, 2012). Individuals obtain information from the external environment, and construct self-cognition and behavior based on it, so as to keep themselves consistent with the external environment (Bandura, 2001). SCT mainly contains three important contents: observational learning, ternary interactive determinism, and self-efficacy theory.

#### Observational learning

SCT suggests that an individual’s behavior is influenced not only by innate factors (e.g., genetics, physiology, etc.) but also by factors acquired by the individual (Bandura, 1986). Among the acquired factors that influence individuals’ behavior, individual observational learning is crucial. Bandura (1986) further states that most of an individual’s behavior is learned by imitation through observation of people, events and objects around them. The behavior of the enterprise will directly have an important role model on the psychology and behavior of the frontline employees. The fulfillment of CSR in tourism enterprises can enable employees to observe the importance and concern of enterprises to various stakeholders. Then employees would regard enterprises’ performance as their code of conducting and adopting behaviors that are beneficial to stakeholders, e.g., SI.

#### Ternary interactive determinism

Triadic interactive determinism holds that the individual’s environment, cognition and behavior do not exist in isolation, and the formation of each of them is determined by their interaction (Bandura, 1986). First, the external environment has a direct impact on individual behavior. Secondly, the internal factors of an individual are mainly composed of cognitive, emotional, physiological and other factors, which can promote or inhibit the behavior of an individual (Bandura, 1986). The fulfillment of CSR will make employees feel the importance and concern of the enterprise to the individual employees and the external society. Then employees’ strong sense of belonging and recognition may be enhanced, thereby promoting employees’ extra-role behaviors, e.g., SI.

#### Self-efficacy

SE is an individual’s subjective cognition and judgment on whether he can complete a certain task (Bandura, 1986). According to SCT, SE has at least four functions: (i) determining people’s choice of activity and their adherence to the activities; (ii) influencing the acquisition of new behaviors and the performance of learned behaviors; (iii) influencing emotions during the activity; and (iv) influencing people’s attitudes in the face of difficulties. A responsible organizational atmosphere will give employees more opportunities for trial and error, which will make them feel more willing to innovate (Bandura, 1986). SI is an extra-role behavior that deviates from the original service standard of the tourism enterprise. Moreover, the result of this behavior is unknown and may produce both positive and negative results. The fulfillment of CSR in tourism enterprises will lead to the perception of employees of the enterprise’ attention to them and customers. Then, such perception will encourage employees to believe that their extra-role behaviors will be supported and understood by the organization. And the employees would believe that they can complete the corresponding service behaviors.

### CSR in the tourism industry in the context of COVID-19

The concept of corporate social responsibility (CSR) was first introduced in 1924 by Oliver Sheldon, who pointed out that the purpose of business cannot be solely for the benefit of shareholders, but that the interests of other stakeholders (including customers, employees, and the public) must be fully considered. Carroll (1999) stated that businesses must not only achieve economic goals within the law, but also meet ethical standards and carry out charitable philanthropic. Based on this, Carroll (1999) constructed the famous CSR pyramid model from four dimensions: economic, legal, ethical, and philanthropic. Subsequently, CSR was expressed as the behavior of businessmen in the pursuit of business interests while complying with social rules and institutions, in line with what the public values pointed to Zhang et al. (2021). Although there is no unified concept of CSR so far, the basic idea of CSR is that enterprises should also make positive contributions to internal (e.g., employees) and external (e.g., customers and the society) stakeholders while pursuing their own economic interests (Shin et al., 2021). Early CSR researches had mostly examined the impact of CSR on corporate performance at the organizational level (Ham and Kim, 2020), but recent researches had gradually shifted to the individual level to discuss the impact of CSR on employees (Ou et al., 2021). These researches focused on how employees perceived, evaluated and reacted to the fulfillment of CSR (Zhang et al., 2021). Li and Fu (2014) stated that employees’ perceptions of CSR were a key factor in their attitudinal and behavioral responses to the corporate.

Compared to other industries, the tourism industry is a service industry as well as a resource-dependent industry, which involves more stakeholders (Henderson, 2007). Therefore, while pursuing economic interests, tourism enterprises need to pay more attention to fulfill their social and environmental responsibilities, especially in the context of COVID-19 (Shin et al., 2021). Mao et al. (2021) empirically analyzed that tourism CSR has an important role in promoting employees’ psychological capital. Shin et al. (2021) verified the impact of hotel CSR on booking behavior and hotel performance. From the perspective of crisis management, Ou et al. (2021) explored the importance of CSR for internal and external stakeholders in different time periods. They argued that a good CSR reduces the stress of internal stakeholders (e.g., employees) to stay on track in the face of crises and gives external stakeholders (e.g., society and suppliers) more confidence. However, compared to general CSR research, relatively little research has been conducted on CSR of tourism enterprises, especially in the context of COVID-19 (Shin et al., 2021).

### Service improvisation

As an important ability of organizations and individuals in the face of uncertain environment (John et al., 2006), improvisation has gradually attracted the attention of various disciplines, especially with the outbreak of COVID-19 at the end of 2019. Secchi et al. (2020) argued that service improvisation (SI) refers to the behavior that frontline employees of service enterprises deviated from established service delivery processes and practices and made immediate responses to unforeseen events with available resources.

It has been documented that SI is driven by a combination of internal and external factors. At the individual level, customer factors are an important driver of SI. From the perspective of value co-creation, customers participate in the production process of service. It is precisely the participation of customers that greatly increases the uncertainty of service, which requires frontline staff to SI (John et al., 2006). In the era of experience economy, customers are no longer satisfied with standardized services, and their needs tend to be personalized. Therefore, frontline employees need to improvise on the original standardized service strategy to meet customers’ personalized service needs (Secchi et al., 2020). In addition, the customer-employee relationship is also an important factor influencing SI. Hultman et al. (2019) pointed out that a good customer-employee relationship enhanced the performance of employee SI. Besides customer factors, the factors of frontline employees themselves can also affect SI. For example, frontline employees’ self-efficacy (Pina e Cunha et al., 2009), service experience (Yeboah Banin et al., 2016), and emotional intelligence (Hill et al., 2017), all influence employees’ SI. At the leadership level, the attitude of managers towards SI affects the subsequent behavior of employees. Leadership styles such as empowering, responsible and innovative promote SI, while leadership styles such as centralized and bureaucratic are important barriers to SI (Turley and O’Donohoe, 2017). At the organizational level, organizational culture (Leybourne, 2009), organizational structure (Pina e Cunha et al., 2009), organizational memory (Nisula and Kianto, 2015), and organizational climate are all important factors that influence SI. In addition, due to the specific nature of the service industry, service scenarios can also have a significant impact on SI (Robson et al., 2015).

SI is not inherently good or bad (Vera and Crossan, 2005), which leads to the fact that it can have both positive and negative outcomes. From the individual level, customers are the direct audience of SI, which means that customer satisfaction is an important result of SI (Secchi et al., 2020). Furthermore, employees are the subject of SI, and having to deal with unexpected service demands in a short period of time is bound to have important effects on the employees themselves, such as time pressure, emotional anxiety, and job satisfaction (Secchi et al., 2020). As SI is the behavior of frontline employees deviating from service standards, its influence on the organization should not be underestimated. On the negative side, SI can have a significant negative impact on an organization’s service performance, corporate image (Lee et al., 2020). On the positive side, it has a catalytic effect on organizational innovation, and organizational knowledge (García-Rosell et al., 2019). However, the existing studies on the effect are mostly theoretical explanations and discussions, lacking empirical tests (Secchi et al., 2020).

### CSR and SI

In the wake of the COVID-19 outbreak, many tourism enterprises have actively pursued their CSR in response to the crisis. The perception and stress of employees in tourism enterprises about the risks associated with diseases affects the mental state and behavior of employees (Vu et al., 2022). According to SCT, the fulfillment of internal social responsibilities by tourism enterprises will make employees feel the care and love of the enterprise, and reduce the pressure on them to face risks. In this way, employees also have enough emotional value to respond to the uncertain service needs of customers, that is, service improvisation. In addition, the fulfillment of external social responsibilities by tourism enterprises sets a good example for employees, and it is easy to enhance employees’ sense of identity and pride in the organization. Therefore, employees will learn from the company, actively consider the difficulties faced by their customers and provide personalized service. Lee et al. (2020) pointed out that tourism enterprises’ CSR fulfillment for frontline employees is conducive to improving the service quality and loyalty of employees to the company. Onyishi et al. (2020) argued that CSR is an extra-role behavior of the company, so the fulfillment of CSR sets a good example to employees, which motivates them to actively engage in extra-role behavior. From the perspective of workplace safety management, Vu et al. (2022) discussed that the fulfillment of CSR can significantly affect the organizational citizenship behavior of employees in the face of COVID-19. During the COVID-19 pandemic, employees are facing greater work pressure and the constant threat of disease (Vaziri et al., 2020). Tourism enterprises should actively fulfill their CSR, such as safety training and salary increases, which can reduce employees’ panic about the epidemic (Hu et al., 2021), so as to ensure employees’ sense of work security, thereby enhancing their organizational citizenship behavior (Vu et al., 2022). Service improvisation as an organizational citizenship behavior, we have reason to believe that CSR also have a positive role in promoting frontline employee SI.

Based on the above analysis, this study puts forward the following hypothesis:

*H1*: CSR has a significant positive impact on SI of frontline employees.

### CSR and SE

Self-efficacy (SE) is essentially a belief in ability or anticipation, a belief in the ability that people have to believe that they can accomplish a particular task (Kim and Beehr, 2017). Many studies have found that leadership style has a significant impact on employees’ SE. Kim and Beehr (2017) pointed out that an empowering leadership style enhanced employees’ autonomy at work and provided a direct contribution to their SE. Khan et al. (2020) suggested that pathological leadership was negatively related to employees’ SE.

Despite the importance of SE, research on the antecedent variables of self-efficacy has grossly neglected the role of external factors in promoting and enhancing SE (Guan and So, 2016), particularly CSR (Latif et al., 2020). According to SCT, individuals can form SE through various social information, including direct experience, observation, and feedback from others. For frontline employees of tourism enterprises in the context of the COVID-19 epidemic, the fulfillment of social responsibility of tourism enterprises is an important source of information (Vu et al., 2022). CSR increases employees’ respect and identification with the company, which in turn influences the development of positive work attitudes (Onyishi et al., 2020), e.g., SE. The fulfillment of tourism CSR will give frontline employees sufficient support and confidence, and they will actively respond to the needs of customers who deviate from service standards without worrying about the company’s penalties and blame. The fulfilment of CSR sets a good example for employees, which makes it easier for them to find meaning in their work (Aguinis and Glavas, 2019). Mao et al. (2021) found that tourism CSR can significantly improve employees’ SE in the face of the COVID-19 epidemic. They confirmed that good corporate social responsibility in tourism enterprises will give frontline employees more understanding and support, and will boost their confidence in facing difficulties.

Based on the above analysis, this study puts forward the following hypothesis:

*H2*: CSR has a significant positive impact on SE of frontline employees.

### SE and SI

In the context of COVID-19, with the increase of service distance, the transformation of service methods, etc., the original service standards can no longer meet the changing situation (Yu et al., 2021), which requires frontline employees of tourism enterprises to actively play SI according to the service situation. SI is essentially an organizational citizenship behavior. The behavior is voluntary and creative by the employee that goes beyond the formal requirements of the employee’s job description, which contributes to the development of the organization. In addition, the outbreak of COVID-19 has also made frontline employees face the constant threat of illness, which has put a huge strain on their bodies and minds. The inapplicability of the original service standards and the threat of disease have brought great uncertainty to the service behavior of frontline employees. This requires frontline employees of tourism enterprises to actively play SI to meet customer needs in the short service contact process (Tang et al., 2020). Existing research confirms that SE can positively influence employee work attitudes, work behaviors and performance (Newman et al., 2014). Individuals with high SE have lower job anxiety and higher self-confidence when dealing with challenges and overcoming unforeseen difficulties (Judge et al., 2007). In the tourism industry, employees with high levels of SE often go beyond the normal job requirements and take the initiative to innovate to solve particular customer problems (Chen et al., 2015), also known as SI (Qiu et al., 2020). A strong sense of SE can enhance a employee’s sense of accomplishment and personal wellbeing (Multon et al., 1991). Employees with high SE may have higher job performance than those with low SE, whereas employees with low self-efficacy may doubt their abilities and avoid difficult tasks (Khan et al., 2020). Tang et al. (2020) confirmed the positive effect of employees’ SE on work behavior in the context of job uncertainty. Ullah S. et al. (2021) also validated the role of SE in promoting employee organizational citizenship. In addition, it has been shown that SE has a positive effect on job performance and career retention (Chuang et al., 2007).

Based on the above analysis, this study puts forward the following hypothesis:

*H3*: SE has a significant positive impact on the SI of frontline employees.

### The mediating role of SI

SCT states that employees’ behavior is influenced by their internal psychological state and external environment. Based on this, some scholars argue that the external environment not only directly affects the behavior of employees, but also affects the behavior of employees through the mediating effect of psychological state (Ullah I. et al., 2021; Zhao and Zhou, 2021). SE, an important positive psychological state, has been shown to play an important mediating role between organizational context and employee behavior (Walumbwa et al., 2011; Yang et al., 2016). Some scholars have explored the mediating role of team efficacy from an organizational level perspective. For instance, Lin et al. (2012) confirmed that CSR indirectly contributes to team performance through team efficacy. Latif et al. (2020) also found the mediating role of team efficacy between CSR and organizational performance. The existing literature also explores the mediating role of SE at the individual level. Peng and Mao (2015) pointed out that the relationship between employee person-job fit and job satisfaction is mediated by SE. Zhang et al. (2020) also confirmed that an individual’s perceived work environment also has an indirect impact on job satisfaction through the mediating role of SE. Kondratowicz et al. (2022) validated the mediating role of SE in the context of COVID-19 between employee job satisfaction and shift in work styles.

Based on the above analysis, this study puts forward the following hypothesis:

*H4*: SE plays a mediating role between the relationship of CSR and SI of frontline employees.

**Figure 1:**
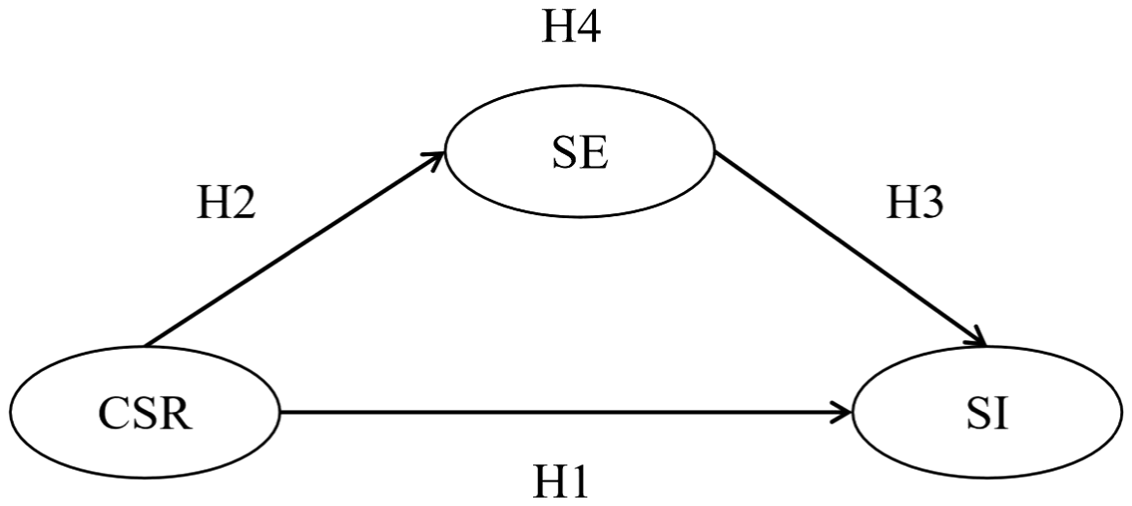
Research framework. CSR, corporate social responsibility; SI, service improvisation; SE, self-efficacy.

To sum up, the following theoretical model is constructed as Figure 1.

## Research method

### Research subjects and data collection

This study focuses on frontline employees of tourism enterprises such as scenic spots above AAA level, and hotels, and tourism agencies with three-star level or above.

This study collected the data from frontline employees of tourism enterprises in Guangzhou, China during three distinct time points (Kim and Kim, 2021). An online survey system was used. To decrease the harmful effects of sampling bias, this study used a random sampling method. Also, this study tried to resolve the limitations embedded in cross-sectional data by collecting the data at three distinct time points.

At Time Point 1, a total of 1,000 questionnaires were distributed to frontline employees, and 714 questionnaires were returned. At Time Point 2, this study sent emails to 714 employees in Time Point 1, and a total of 537 questionnaires were collected. At Time Point 3, this study sent emails to 537 employees in Time Point 2, and a total of 405 questionnaires were collected. The interval between each time point was about 2 weeks. The features of the respondents are described in Table 1.

**Table 1:** Descriptive features of the sample (*N* = 405).

Characteristic		Percent	Effective percentage	Cumulative percentage
Enterprise type	Hotel	151	37.3	37.3
	Tourism agency	136	33.5	70.8
	Scenic spot	118	29.2	100
Gender	Male	168	41.5	41.5
	Female	237	58.5	100
Age	Below 20	107	26.3	26.3
	Between 20 and 25	187	46.1	72.4
	Between 26 and 31	112	27.6	100
Working years	Below 1	111	27.5	27.5
	Between 1 and 3	188	46.3	73.8
	Between 4 and 6	94	23.2	97
	7 and above	12	3	100
Education	Below junior college	157	38.7	38.7
	Junior college	163	40.3	79
	Bachelor’s degree or above	85	21	100

### Instruments

This study developed a questionnaire with five sections. The first to fourth sections are designed to measure CSR, SE, and SI, respectively. The fifth section investigates frontline employees’ demographic traits (e.g., enterprise type, gender, age, work years, and education). The scales are designed according to the research objectives and the existing literature. A total of 24 items were scored on a five-point Likert scale, ranging from 1 (strongly disagree) to 5 (strongly agree).

#### CSR (time point 1, gathered from frontline employees)

At Time Point 1, this study used five items from Chua et al. (2020) to measure CSR. Sample items are “After the outbreak of the COVID-19 pandemic, our tourism enterprise can handle the relationship with partners well,” “After the outbreak of the COVID-19 pandemic, our tourism enterprise actively fulfilled its responsibility to the environment,” and “After the outbreak of the COVID-19 pandemic, our tourism enterprise can pay attention to the physical and mental health and professional development of employees.”

#### SE (time point 2, gathered from frontline employees)

At Time Point 2, this study used four items from Liu et al. (2014) to measure SE. Sample items are “I think I am good at coming up with new ideas,” and “I have confidence in my ability to creatively solve problems.”

#### SI (time point 3, gathered from frontline employees)

At Time Point 3, this study used eleven items from Secchi et al. (2020) to measure SI. Sample items are “I often find new service methods to meet specific customer requirements,” “In the process of serving customers, I often respond immediately to unexpected problems,” and “I often get information from many different sources in response to customer requests.”

### Data analysis

The analysis was based on the structural equation model (SEM). SEM allows independent variables and dependent variables to contain measurement errors, and these errors can be eliminated through the measurement equation between the explicit and implicit variables (Hair et al., 2014). SEM combines two statistical techniques, factor analysis, and path analysis, which integrate factor analysis and multiple regression analysis. It can simultaneously measure and analyze multiple independent relationships. The assessment of a model using SEM generally follows a two-step process, namely, assessments of the measurement model and the structural model (Hair et al., 2014). Assessment of the measurement model entails the evaluation of the validity and reliability centered on the model’s latent variables (LVs). This evaluation involves the assessment of the relationships between the LVs and their associated items. The assessment of the structural model is concerned with the relationships between LVs (Hair et al., 2014). Additionally, this study adopts a bootstrapping method to verify the mediating role of SE.

## Results

### Measurement model

#### Descriptive statistical analysis

SPSS 25 was used to perform the descriptive statistical analysis of each measurement scale (Table 2). The standard deviations of the measurement items of SI, SE, and CSR are relatively stable. The skewness and kurtosis are also relatively stable, and the absolute values of the skewness and kurtosis are all less than 3, indicating that the scores of the surveyed objects are highly effective and stable (Lu et al., 2020).

**Table 2:** Descriptive statistics.

Scale	Item No.	Mean	Std. deviation	Skewness		Kurtosis	
				Statistic	Std. error	Statistic	Std. error
SI	SI1	3.43	0.872	0.007	0.121	−0.378	0.242
	SI2	3.3	0.897	0.142	0.121	−0.494	0.242
	SI3	3.22	0.943	−0.22	0.121	−0.384	0.242
	SI4	3.61	0.803	−0.189	0.121	−0.109	0.242
	SI5	3.96	0.75	−0.423	0.121	0.001	0.242
	SI6	3.17	0.794	0.109	0.121	−0.34	0.242
	SI7	3.89	0.73	−0.175	0.121	−0.359	0.242
	SI8	3.03	0.941	0.343	0.121	−0.201	0.242
	SI9	2.92	1.028	0.361	0.121	−0.521	0.242
	SI10	2.93	0.94	0.528	0.121	0.103	0.242
	SI11	2.82	0.947	0.515	0.121	0.306	0.242
SE	SE1	3.55	0.735	0.227	0.121	−0.353	0.242
	SE2	3.76	0.733	−0.152	0.121	−0.246	0.242
	SE3	4.07	0.766	−0.388	0.121	−0.482	0.242
	SE4	3.98	0.748	−0.111	0.121	−0.836	0.242
CSR	CSR1	3.87	0.689	−0.142	0.121	−0.206	0.242
	CSR2	3.66	0.709	0.102	0.121	−0.385	0.242
	CSR3	3.59	0.714	−0.033	0.121	0.216	0.242
	CSR4	3.69	0.913	−0.423	0.121	−0.234	0.242
	CSR5	3.74	0.753	−0.271	0.121	0.407	0.242

CSR, corporate social responsibility; SI, service improvisation; SE, self-efficacy.

#### Reliability and validity

The assessment of the measurement model involves an evaluation of reliability and validity. Reliability is mainly measured from two aspects: corrected item total correlation (CITC) and Cronbach’s α (Hair et al., 2014). Validity in turn comprises two main types: convergent and discriminant validity. Convergent validity is often assessed by way of two key coefficients [56]: the composite reliability (CR) and average variance extracted (AVE). In assessing a model’s convergent validity, the loading of each indicator on its associated LV must be calculated and compared to a threshold. Generally, the loading should be higher than 0.7 for validity to be considered acceptable.

According to Preacher and Hayes (2008), the reliability of each scale can be ensured by calculating the value of CITC and Cronbach’s α after deleting the item. Table 3 revealed that Cronbach’s α values for all variables exceeded the minimum threshold level of 0.70, namely 0.820, 0.842, and 0.909. The CITC of each item is higher than 0.5, and the value of each CITC is also less than the Cronbach’s α after deleting the item. Therefore, it indicates the acceptable reliability of all variables used in this study (Preacher and Hayes, 2008).

**Table 3:** Reliability analysis.

	CITC	Cronbach’s α after deleting the item	Cronbach’s α
CSR	0.593	0.791	0.820
	0.658	0.772	
	0.638	0.777	
	0.586	0.800	
	0.614	0.784	
SE	0.663	0.762	0.842
	0.568	0.845	
	0.694	0.792	
	0.686	0.796	
SI	0.625	0.902	0.909
	0.659	0.900	
	0.688	0.899	
	0.609	0.903	
	0.620	0.903	
	0.664	0.900	
	0.617	0.903	
	0.668	0.900	
	0.682	0.900	
	0.686	0.899	
	0.706	0.898	

CSR, corporate social responsibility; SI, service improvisation; SE, self-efficacy.

Table 4 indicates that the CRs for all the LVs in the measurement model exceeded 0.7, namely 0.8385, 0.8471, and 0.8721. It shows that the measurement model presents acceptable composite reliability. In addition to the previously discussed criteria for convergent validity, the AVEs of the LVs should also be higher than 0.5 for their convergent validity to be considered acceptable. Table 4 reveals that AVE for all factors exceeded the minimum threshold value of 0.50, namely 0.512, 0.5830, and 0.6945. It indicates the convergent validity of all variables are acceptable.

**Table 4:** The results of the confirmatory factor analysis.

Latent variable		No.	Factor loading	*R^2^*	CR	AVE
CSR					0.8385	0.512
		CSR1	0.66	0.44		
		CSR2	0.84	0.70		
		CSR3	0.73	0.54		
		CSR4	0.69	0.48		
		CSR5	0.64	0.41		
SE				0.10	0.8471	0.5830
		SE1	0.84	0.70		
		SE2	0.64	0.41		
		SE3	0.78	0.61		
		SE4	0.78	0.60		
SI				0.57	0.8721	0.6945
		SI1	0.77	0.59		
		SI2	0.83	0.69		
		SI3	0.85	0.72		
		SI4	0.75	0.56		
		SI5	0.77	0.60		
		SI6	0.65	0.42		
		SI7	0.77	0.60		
		SI8	0.75	0.56		
		SI9	0.77	0.59		
		SI10	0.81	0.65		
		SI11	0.84	0.71		

CSR, corporate social responsibility; SI, service improvisation; SE, self-efficacy.

Table 5 shows that the correlation coefficients between the three variables are 0.273, 0.426, and 0.583, and the *p*-values of each correlation coefficient are all less than 0.01. In addition, the square root values of AVE are 0.716, 0.764, 0.833, respectively, which are higher than all the correlation coefficient values, so the three variables have satisfactory discriminant validity (Preacher and Hayes, 2008).

**Table 5:** Correlation coefficient analysis.

**Latent variable**	Mean	S.D.	CSR	SE	SI
CSR	3.710	0.580	**0.716**		
SE	3.839	0.614	0.273**	**0.764**	
SI	3.299	0.638	0.426**	0.583**	**0.833**

CSR, corporate social responsibility; SI, service improvisation; SE, self-efficacy; ** = *p* < 0.01; The numbers in bold on the diagonal line represent the square root of the AVE.

### Structural model

The structural model fit was estimated using indices including *χ*^2^/df, RMSEA, GFI, IFI, CFI, TLI (Preacher and Hayes, 2008). When the value of *χ*^2^/df is between 1 and 3, the model has a simple adaptation degree. The standard values of GFI, IFI, TLI, and CFI are all above 0.9, and the standard value of RMSEA is lower than 0.05 (good fit) and less than 0.08 (suitable). Table 6 shows the indexes of this study’s model fitness. From these results, the structural model of this study has a good degree of fitness.

**Table 6:** Fitness index.

*χ^2^*	*χ^2^*/df	SRMR	RMSEA	GFI	CFI	IFI	TLI
445.674	1.165	0.043	0.045	0.915	0.959	0.960	0.954

Some scholars have pointed out that *R*^2^ can represent the explanatory effect of the structural model. When the *R*^2^ value is greater than 0.09, it indicates that the structural model has a good explanatory effect. According to Table 7, in the structural model of this study, the *R*^2^ of SE and SI are 0.10 and 0.57, respectively, indicating that the model of this study has a good explanatory effect.

**Table 7:** Path parameters.

Hypothesis relationship	Path relationship	Estimate	S.E.	C.R.(t)	*p*	*β*	*R^2^*
H1	CSR → SI	0.314	0.063	4.984	***	0.285	**0.57**
H2	CSR → SE	0.398	0.074	5.404	***	0.320	**0.10**
H3	SE → SI	0.485	0.055	8.875	***	0.548	**0.57**

CSR, corporate social responsibility; SI, service improvisation; SE, self-efficacy; *** = *p* < 0.001.

As shown in Table 7, CSR has a significant positive effect on SI (*β* = 0.285, *p* < 0.001) and SE (*β* = 0.320, *p* < 0.001). Therefore, Hypotheses H1 and H2 are supported. SE has a significant positive effect on SI (*β* = 0.548, *p* < 0.001), so Hypothesis H3 is also supported.

### Mediation test

This study used Bootstrapping method to test the mediating effect following the existing literature (Preacher and Hayes, 2008). When verifying the mediating effect by the Bootstrapping method, the main reference standard is the confidence interval of the indirect effect. If the upper and lower limits of the confidence interval are both higher than 0 or both lower than 0, that is, if 0 is not included, it indicates that the indirect effect exists.

Table 8 shows that the SE of frontline employees in tourism enterprises has a significant mediating effect on the relationship between CSR and SI (confidence interval is 0.02 to 0.07, which does not straddle 0; Preacher and Hayes, 2008), and the indirect effect is 0.14, which means that Hypothesis 4 in this study is partially supported.

**Table 8:** Confidence interval.

	Effect	Boot SE	Boot LLCI	Boot ULCI
Total effect	0.47	0.05	0.37	0.57
Direct effect	0.28	0.04	0.20	0.37
Indirect effect (SE)	0.14	0.03	0.09	0.19

SE, self-efficacy.

## Discussion

From the perspective of SCT, this study discusses the influencing mechanism of tourism CSR on SI of frontline employees in the context of COVID-19 and explores the midiating role of SE in the relationship. Through the collection and analysis of data from frontline employees at three distinct time points, this study find that CSR has a positive impact on the SI and SE of frontline employees. Furthermore, SE, as an important psychological state, can not only directly promote the SI of frontline employees, but also play a partially mediating role in the relationship between CSR and SI. In a word, this study initially explores the relationship between CSR and SI, and discusses SE to open the “black box” in the tourism industry in the context of COVID-19.

First, we have verified that tourism CSR can directly promote the SI and SE of frontline employees, which is consistent with SCT (Tuan, 2018). According to SCT, the external environment can have a significant impact on an individual’s psychological state and behavioral orientation. That is to say, in the face of crisis, the strategy of tourism enterprises will not only directly affect the performance of enterprises, but also have an impact on the psychological state and service behavior of employees. In the context of COVID-19, the fulfillment of CSR has become an important strategy for tourism enterprises to cope with the crisis. However, the effectiveness of tourism CSR is still worth discussing. Through the study of frontline employees of Chinese tourism enterprises, this study confirms that the fulfillment of tourism CSR is conducive to enhancing the SE and SI of frontline employees, which is similar to previous research (Newman et al., 2014; Gond et al., 2017). This further verifies that the fulfillment of tourism CSR has a certain effect on responding to the crisis. In the context of the COVID-19 pandemic, the fulfillment of tourism CSR not only directly give more care and protection to frontline employees, but also indirectly set a positive example for employees. Therefore, employees will identify more with the enterprise and actively learn from the company. In this way, when they face uncertain needs of customers, they will be more confident in themselves and the enterprise, and then promote SE and SI.

Second, we confirmed that frontline employees’ SE has a positive impact on their SI, which is coherent amid the preceding literature (Hmieleski and Corbett, 2008). According to SCT, individuals choose their behavior through judgments about the way they behave (Huang, 2017). Individuals with high self-efficacy will have strong confidence in their abilities and motivation to act on the actions they choose. SI is a stressful action of an individual to an unknown problem in the context of complex mutations. This immediate action is based on the individual’s self-confidence and good self-perception of his own abilities. Good psychological perception is a powerful motivator for employees to produce improvisational behaviors (Ullah I. et al., 2021). Employees with low self-efficacy who are not confident in their abilities will take a conservative approach to difficulties. On the contrary, employees with high self-efficacy will adopt active and flexible solutions to unexpected problems, so as to meet the needs of self-realization. In the context of the COVID-19 pandemic, frontline employees face more uncertainty. Employees with a high sense of self-efficacy are able to actively face the pressures of the COVID-19 pandemic to better serve their customers in uncertainty situation.

Third, this study found that SE plays a partial mediating role between the relationship of CSR and SI. This finding is consistent with previous research (Ullah I. et al., 2021; Zhao and Zhou, 2021). According to SCT, the external environment affects the behavior of employees through their psychological state. Our research also further confirms SCT. In the context of the COVID-19 pandemic, the fulfillment of tourism CSR can give employees more care and support. In this way, frontline employees will have a higher level of self-efficacy in the face of the pressure of the COVID-19 epidemic. They believe they are capable enough to deal with the dilemma they face. Therefore, they will actively adjust their service strategies according to different service needs to cope with the uncertainty caused by the COVID-19 pandemic.

## Conclusion

### Theoretical contribution

First, this study expands on the study of CSR. On the one hand, this study enriches the study of micro-CSR. Existing CSR research focuses more on the corporate level, and relatively less on the employee level (Ibarnia et al., 2020). From the perspective of micro-CSR, this study explores the influencing mechanism of CSR fulfillment on the psychological state and behavior orientation of frontline employees, and further expands the research on CSR. On the other hand, this study confirms the effectiveness of CSR in tourism companies’ response to COVID-19. CSR has long been debated about its effectiveness in responding to crises (Aguinis et al., 2020). After the outbreak of the new crown epidemic, although many tourism companies are actively fulfilling their corporate responsibilities (Ham and Kim, 2020), there is still no conclusion on whether the fulfillment of corporate social responsibility can help enterprises survive the crisis. This study focuses on frontline employees of Chinese tourism enterprises and confirms that in the context of the COVID-19 pandemic, tourism CSR has a positive impact on the psychological state and behavior of frontline employees. The fulfillment of tourism CSR can not only enhance the SE of employees in the face of crisis, but also encourage employees to actively adopt SI to meet customer needs. This further verifies the effectiveness of CSR response to COVID-19.

Second, this study enriches the study of SCT. On the one hand, this study extends the study of SE in the context of crisis. As a positive psychological state, SE is an assessment and recognition of employees’ own abilities (Bandura, 1986). However, there has been a lack of systematic discussion about whether employees can maintain a high level of SE in times of crisis (Shao et al., 2019). Taking the COVID-19 pandemic as the research background and frontline employees of tourism enterprises as the research object, this study systematically explores the causes and consequences of SE of frontline employees in the face of difficulties such as disease threats and uncertain needs. On the other hand, this study enriches the antecedent and consequence variables of SE. This study introduced CSR as an antecedent variable, introduced SI as an outcome variable, and ultimately verified that SE has a significant mediating role on the relationship between CSR and SI.

Thirdly, this study further enriches the study of improvisation. On the one hand, this study expands the research context. As a high-contact industry, the tourism industry has a high degree of uncertainty in the demand of tourists, which is consistent with the connotation of the concept of improvisation (John et al., 2006). However, most of the existing improvisation research is based on manufacturing and high-tech industries, ignoring the attention and discussion of the tourism industry (Secchi et al., 2020). Taking Chinese tourism companies as a case study, this study systematically explored the improvisation of frontline employees in the face of uncertainty caused by the COVID-19 pandemic. On the other hand, this study enriches the research on the influencing mechanism of SI. Improvisation is an important behavior to deal with uncertainty, and the research on it is more about theoretical derivation and case analysis, and lacks empirical research (Secchi et al., 2020). In the context of the COVID-19 pandemic, this study systematically explored the influencing mechanism of tourism CSR on SI of frontline employees, and verified the mediating role of SE on the relationship between CSR and SI.

### Practical implications

Tourism enterprises should actively fulfill their social responsibilities to customers. In the post-pandemic era, tourism enterprises should actively disclose and update the changes in the pandemic situation in the local city, the weather conditions in recent days, and the health of the customers they receive. Such strategies help to ensure that customers have a safe consumption environment. Tourism enterprises should also actively perform disinfection and epidemic prevention work. For example, they should provide customers with disinfection protection kits for free or at low prices. Besides, tourism enterprises should actively take care of customers’ emotions and special circumstances. For example, when customers are unable to travel or consume due to their physical conditions, tourism enterprises should deal with these problems according to the specific circumstances and formulate clear service procedures. They should try to avoid cumbersome service processes.

Tourism enterprises should also actively fulfill their responsibilities to the public. First, tourism enterprises should rely on the characteristics of industries that are more relevant (e.g., food, lodging, tourism, shopping, and entertainment) to provide the public with more comprehensive and detailed pandemic consultation. Second, tourism enterprises should provide corresponding free or discounted services to individuals that have made important contributions to pandemic prevention and should simplify or clarify the redemption process as much as possible. In addition, tourism enterprises may provide more job opportunities, or materials to families and individuals that have been greatly influenced by the pandemic. For example, hotels can donate some bedding and toiletries and provide some service training.

Tourism enterprises should establish a people-oriented corporate culture. Frontline employees are often at the lowest level of tourism enterprises. They have lower education, lower wages, and lower social status. However, they are the bridge between tourism enterprises and customers. Therefore, tourism enterprises should actively take care of the emotional needs of employees and establish a people-oriented corporate culture.

First, in terms of employee incentives, tourism enterprises should make fair and reasonable arrangements for frontline employees based on their working performance and ability. For example, some employees pointed out that *“My ability is completely capable of being a supervisor. However, to stabilize and take care of the emotions of the old employees, the enterprises gave the position to the old employees.”* This can easily lead to employees’ psychological imbalance, negative emotions, non-active work, and resignation. Second, in terms of employee care, tourism enterprises should set up a special psychological counseling department to guide and manage the emotions of frontline employees, thereby reducing the possibility of their emotional exhaustion. Tourism enterprises should reduce the intensity of punishment, give more recognition and care to frontline employees, stimulate their intrinsic motivation, and help them realize their value. Tourism enterprises may also set up employee clubs, organize birthday parties, sports activities, employee networking, etc., to strengthen communication and exchanges between employers and employees. Third, in terms of customer relations, due to the uneven quality of customers and the uncertainty of customer needs, tourism enterprises should actively protect the interests of frontline employees, especially when frontline employees are improperly treated by customers or unreasonable service needs. Enterprises must actively protect the interests of frontline employees and ensure their physical and mental safety.

## Limitations and suggestions for future research

This study merely delved into the relationship among CSR, SE and SI from frontline employees’ perspectives in tourism enterprises. Therefore, future research should take one step further from the following aspects. First, different types of employees can be investigated, and horizontal comparative analysis should be conducted in future research. Second, future research may collect the data from the customer perspective to obtain more objective research results. In this study, in-depth interviews and questionnaires are mainly used for data collection. Both two data collection methods are highly subjective and are easily affected by factors such as the surrounding environment, one’s emotional state, memory bias, etc. Therefore, future research should adopt a more comprehensive and well-designed approach to collect the data.

## Data availability statement

The original contributions presented in the study are included in the article/supplementary material, further inquiries can be directed to the corresponding author.

## Ethics statement

The studies involving human participants were reviewed and approved by the Ethics and Academic Committee of Zhoukou Normal University. The patients/participants provided their written informed consent to participate in this study.

## Author contributions

XZ contributed to the literature review and supervision, wrote the original draft, and provided fund support. SZ analyzed and interpreted the data, wrote the original draft, edited the manuscript, and provided fund support. MW contributed to the literature review and supervision, analyzed and interpreted the data, wrote the original draft, and edited the manuscript. All authors have read and agreed to the published version of the manuscript.

## Funding

This work was supported by 2023 General Project on Humanities and Social Sciences Research in Henan Universities (Research on ecological security of wetland tourism area in Henan Province from the perspective of community residents, grant no. 2023-ZDJH-058), Henan Philosophy and Social Sciences Planning Project (grant no. 2022CJ177), and Tertiary Education Scientific research project of Guangzhou Municipal Education Bureau (grant no. 202235337).

## Conflict of interest

The authors declare that the research was conducted in the absence of any commercial or financial relationships that could be construed as a potential conflict of interest.

## Publisher’s note

All claims expressed in this article are solely those of the authors and do not necessarily represent those of their affiliated organizations, or those of the publisher, the editors and the reviewers. Any product that may be evaluated in this article, or claim that may be made by its manufacturer, is not guaranteed or endorsed by the publisher.
